# Supplementary material for: MetaRibo-Seq measures translation in microbiomes
Source: Nat Commun. 2020 Jun 29;11:3268. doi: 10.1038/s41467-020-17081-z (PMC7324362; doi:10.1038/s41467-020-17081-z)
Supplement: Supplementary file 10 — Supplementary Data 7 [file 41467_2020_17081_MOESM10_ESM.zip › File2/Confidence_High_Taxonomy/323989_out.krona.html]

Javascript must be enabled to view this page.

members
magnitude
magnitudeUnassigned
count
unassigned
taxon
rank

323989\_out

15

superkingdom
2
15

phylum
201174
1

class
1
1760

order
2037
1

2049
1
family

genus
1654
1

1
1655
species

SRS143216\_contig\_number\_33116

14
1239
phylum

14
909932
class

14
909929
order

1843491
14
family

genus
1
970

SRS013705\_contig\_number\_14381
14


SRS016969\_contig\_number\_contig-100\_789.265091SRS017757\_contig\_number\_32038SRS018739\_contig\_number\_13819SRS023595\_contig\_number\_contig-100\_4074.315566SRS097895\_contig\_number\_21084SRS142787\_contig\_number\_24439SRS143062\_contig\_number\_10633SRS144124\_contig\_number\_26537SRS144300\_contig\_number\_15118SRS144560\_contig\_number\_contig-100\_673.563410SRS147547\_contig\_number\_8819SRS148472\_contig\_number\_41340
species
69823
12

712538
1

SRS148150\_contig\_number\_10561
species
